# The nonsense-mediated mRNA decay (NMD) pathway differentially regulates *COX17, COX19* and *COX23* mRNAs

**DOI:** 10.1007/s00294-018-0892-y

**Published:** 2018-10-13

**Authors:** Kaitlin Murtha, Munok Hwang, Megan C. Peccarelli, Taylor D. Scott, Bessie W. Kebaara

**Affiliations:** 1 Department of Biology, Baylor University, One Bear Place #97388, Waco, TX 76798 USA; 2 University of Wisconsin-Madison, Madison, WI 53706 USA

**Keywords:** *Saccharomyces cerevisiae*, mRNA, mRNA decay, Mitochondrial copper homeostasis, Nonsense-mediated mRNA decay

## Abstract

The differential regulation of *COX17, COX19* and *COX23* mRNAs by the nonsense-mediated mRNA decay (NMD) pathway was investigated. The NMD pathway regulates mRNAs that aberrantly terminate translation. This includes mRNAs harboring premature translation termination codons and natural mRNAs. Most natural mRNAs regulated by NMD encode fully functional proteins involved in various cellular processes. However, the cause and targeting of most of these mRNAs by the pathway is not understood. Analysis of a set of mRNAs involved in copper homeostasis showed that a subset of these mRNAs function in mitochondrial copper homeostasis. Here, we examined the regulation of *COX17, COX19* and *COX23* mRNAs by NMD. These mRNAs encode homologous mitochondrial proteins involved in metallation of cytochrome *c* oxidase. We found that *COX17, COX19* and *COX23* mRNAs are differentially regulated by NMD depending on environmental copper levels. A long 3′-UTR contributes to the direct regulation of *COX19* mRNA by the pathway. Alternatively, *COX23* mRNA contains a long 3′-UTR, but is indirectly regulated by the pathway under two conditions tested here. Analysis of the functionality of the NMD targeting features in *COX23* mRNA showed that the *COX23* 3′-UTR is sufficient to trigger NMD. The regulation of mRNAs involved in mitochondrial copper metabolism by NMD is physiologically significant because excess copper enhances growth of NMD mutants on a non-fermentable carbon source. These findings suggest that regulation of mRNAs encoding homologous proteins by NMD can be differential depending on environmental copper levels. Furthermore, these findings suggest copper ion homeostatic mechanisms in the mitochondria occur at the mRNA level via the NMD pathway.

## Introduction

The nonsense-mediated mRNA decay (NMD) pathway is a highly conserved mRNA degradation pathway found in all tested eukaryotes from yeast to humans. In *Saccharomyces cerevisiae*, ~ 5–10% of the transcriptome is affected when NMD is non-functional (He et al. 2003; Guan et al. 2006; Celik et al. 2017). Similar results were observed in studies involving *Drosophila melanogaster* and humans (Mendell et al. 2004; Rehwinkel et al. 2005; Wittmann et al. 2006). Upf1p, Upf2p and Upf3p are three core NMD factors that are required for a functional NMD pathway in all organisms.

NMD was first identified as a pathway that degrades premature termination codon (PTC) containing mRNAs, therefore preventing the synthesis of potentially harmful truncated proteins. NMD is now also recognized as a pathway that degrades natural mRNAs that encode fully functional proteins. Thus, NMD plays dual roles, one in mRNA surveillance and a second in regulation of gene expression. NMD-mediated degradation of natural mRNAs has been observed in diverse organisms including *S. cerevisiae, D. melanogaster, Caenorhabditis elegans*, plants and humans.

Natural mRNAs regulated by NMD are either direct or indirect targets. Direct NMD targets have significantly altered mRNA decay rates in cells with a functional versus non-functional NMD pathway. Conversely, indirect NMD targets have comparable decay rates in cells with a functional and non-functional NMD pathway. mRNAs that are direct targets typically have a variety of recognized NMD-inducing features in eukaryotes. In *S. cerevisiae*, these features include translated upstream open reading frames (uORF) (He et al. 2003; Gaba et al. 2005; Guan et al. 2006; Johansson and Jacobson 2010), out of frame initiation of translation (also known as “leaky scanning”) (Welch and Jacobson 1999; Guan et al. 2006), inefficient and alternatively spliced pre-mRNAs (He et al. 1993; Guan et al. 2006), ribosomal frameshift signals (Belew et al. 2011; Celik et al. 2017), and atypically long 3′-untranslated regions (UTR) (Guisbert et al. 2007; Kebaara and Atkin 2009; Deliz-Aguirre et al. 2011). In addition, regulation of natural mRNAs by NMD can be growth condition specific. These observations have led to the hypothesis that these NMD targeting features can function in specific cellular context and environmental conditions (Peccarelli et al. 2016).

Our previous studies found that *S. cerevisiae* cells with an inactive NMD pathway are more tolerant of toxic copper levels (Deliz-Aguirre et al. 2011). To examine this phenotype further, we investigated a subset of mRNAs involved in copper homeostasis to gain insight into their regulation by NMD. Understanding the role NMD plays in copper homeostasis is important because copper is essential for cellular function but can be toxic in excess. Additionally, understanding copper homeostatic mechanisms in yeast will provide insight into the regulatory mechanisms used in other organisms as these homeostatic mechanisms have been conserved throughout evolution (De Freitas et al. 2003).

Analysis of a set of mRNAs involved in copper homeostasis revealed that a subset of these mRNAs function in mitochondrial copper utilization. Copper is an essential metal in the mitochondria for the functions of cytochrome *c* oxidase (CcO), and copper, zinc-superoxide dismutase (Sod1). The mRNAs we examined here are involved with CcO metallation. Cox17p was initially identified as an *S. cerevisiae* CcO assembly mutant that was unable to carry out respiratory growth in the presence of CcO components. Subsequently, Cox17p was found to be a copper-binding protein that delivers copper to CcO through two copper-binding intermembrane space-associated proteins Sco1 and Cox11. Mice lacking *COX17* function show impaired CcO activity and die early in utero. Cox19p and Cox23p are homologs of Cox17p and are required for the assembly of CcO and for respiration. Cox17p, Cox19p and Cox23p contain twin cysteine-x_9_-cysteine (twin Cx_9_C) motifs and all have human orthologues (Longen et al. 2009). Cox23p is a mitochondrial intermembrane space protein that functions in mitochondrial copper homoeostasis. Cox19p is found in the cytosol and the mitochondrial intermembrane space and is required for the assembly of CcO.

Since *COX17, COX19* and *COX23* mRNAs encode functionally related proteins, we examined the extent to which *COX17* mRNA is an NMD target based on our earlier observation that *COX19* and *COX23* mRNAs are NMD targets in rich media. We previously found that *COX19* and *COX23* mRNAs have atypically long 3′-UTRs, but are differentially regulated by the pathway in rich media. The *COX19* mRNAs long 3′-UTR contributes to the degradation of the mRNA by NMD. Alternatively, despite having a long 3′-UTR, *COX23* mRNA is indirectly regulated by the pathway in rich media (Peccarelli et al. 2016). Since these mRNAs have NMD targeting features, the pathway could directly regulate them in select environmental conditions. For example, we previously found that *MAC1* mRNA was directly regulated by NMD in rich media, but not regulated under low copper conditions, when the Mac1p is required (Peccarelli et al. 2016). Thus, we examined whether environmental copper levels affect the NMD-mediated regulation of these three mRNAs. Additionally, we examined the functionality of the NMD-targeting features in *COX23* mRNA. We investigated the extent to which the long 3′-UTR is sufficient to target *COX23* mRNA to NMD by replacing it with the short *CYC1* 3′-UTR which known to not trigger NMD. This enabled us to understand if the *COX23* mRNA 3′-UTR was too short to activate NMD, is present in an incorrect context in *COX23*, or whether the NMD targeting features in *COX23* function only under specific environmental conditions not examined here.

NMD mutants have respiratory impairments when grown on non-fermentable carbon sources. This growth impairment could be due to the accumulation of aberrant products interfering with respiratory function or altered expression of mRNAs and consequently proteins involved in mitochondrial copper homeostasis. Interestingly, yeast strains lacking *COX17, COX19* and *COX23* show impaired growth on non-fermentable carbon sources (Longen et al. 2009). In addition, this phenotype is observed when mitochondrial copper homeostasis is misregulated. We examined the extent to which the respiratory defect observed in NMD mutants is due to an imbalance in copper homeostatic mechanisms in the mitochondria. NMD mutants grown on lactate, a non-fermentable carbon source, under normal copper conditions, have impaired growth relative to wild-type cells. Interestingly, growth under the same conditions with media supplemented with excess copper recovered the growth defect of the NMD mutants. Additionally, we found that overexpression of *COX19* results in increased tolerance of wild-type cells to elevated copper levels.

## Materials and methods

### Yeast strains

All *S. cerevisiae* strains and genotypes used in this study are listed in Table 1.

**Table 1:** *Saccharomyces cerevisiae* strains used in this study

Yeast strain	Genotype	Source
W303	*a, ade2-1, ura3-1, his3-11,15, trp1-1, leu2-3,112, can1-101*	Wente et al. (1992)
AAY320	*a, ade2-1, ura3-1, his3-11,15, trp1-1, leu2-3,112, can1-100, UPF1::URA3 (upf-Δ2)*	Kebaara et al. (2003)
AAY327	*a, ade2-1, ura3-1, his3-11,15, trp1-1, leu2-3,112, can1-100, UPF1::TRP (upf1-Δ6)*	Kebaara et al. (2003)
AAY334	*a, ADE2, ura3-1 or ura3-52, his3-52, his3-11,15, trp1-1, leu2-3,112, rpb1-1*	Kebaara et al. (2003)
AAY335	*a, ADE2, ura3-1 or ura3-52, his3-52, his3-11,15, trp1-1, leu2-3,112, rpb1-1, upf1-Δ2 (URA3)*	Kebaara et al. (2003)
HFY1300	*MATα ade2-1 ura3-1 his3-11,15 trp1-1 leu2-3,112 trp1-1can1-100 UPF1 nmd2::HIS3 UPF3*	He and Jacobson (1995)
HFY861	*MATa ade2-1 ura3-1 his3- 11,15 trp1-1 leu2-3,112 trp1-1can1-100 UPF1 NMD2 upf3::HIS3*	He et al. (1997)

### Growth of yeast strains

Yeast strains were maintained and grown using standard techniques (Ausubel et al. 1998). For analysis of *COX17, COX19* and *COX23* mRNAs under low copper conditions, wild-type and NMD mutant yeast cells were grown in low copper complete minimal (CM) media. This media contained yeast nitrogen base without copper (YNB–CuSO_4_–FeCl_3_) and 100 µM bathocuproinedisulfonic acid (Sigma–Aldrich). Glassware used in these experiments was soaked in 10% nitric acid overnight to remove trace amounts of copper. All yeast cells used for low-copper northerns were initially grown to saturation in CM media and then subcultured into copper-deficient media in acid-washed glassware.

To analyze *COX17, COX19* and *COX23* mRNAs under high copper conditions, wild-type and NMD mutant yeast cells were grown in CM media supplemented with 100 µM copper (high copper media). As with the low copper conditions, the yeast cells were first grown to saturation in complete minimal media then subcultured into media supplemented with 100 µM copper.

### RNA methods

For all mRNA steady-states and half-life experiments total *S. cerevisiae* RNA was used. Yeast cells cultured in the different conditions described above were harvested at mid-log phase as described in Peccarelli and Kebaara (2014). Total RNA was extracted from harvested cells using the hot phenol method. 15 µg of total RNA was resolved on 1.0% agarose-formaldehyde gels for all steady-state and half-life northerns. Then, the RNA was transferred to GeneScreen Plus^®^ (PerkinElmer, Boston, MA, USA) nylon membranes using the NorthernMax™ Complete Northern Blotting kit (Thermo Fisher Scientific, Carlsbad, CA, USA) transfer protocol. Northern blots were probed with oligolabeled DNA probes that were labeled with [α-^32^P] dCTP using the RadPrime DNA Labeling System (Thermo Fisher Scientific, Carlsbad, CA, USA). All DNA probes were generated by PCR. Northern blots were phosphorImaged™ using a Typhoon Phosphorimager (Amersham Pharmacia Biotech, Inc.).

For low copper controls, all northerns with RNA extracted from yeast cells grown under low copper conditions were probed with *CTR1. CTR1* encodes a high affinity copper transporter of the plasma membrane. Diminished copper levels result in increased *CTR1* expression. For high copper controls, all northerns with RNA extracted from yeast cells grown in 100 µM copper were probed with *CUP1. CUP1* encodes a metallothionein that binds copper. The *CUP1* gene is induced by the Ace1 transcription factor when yeast cells are exposed to elevated copper levels. Increases in copper levels result in increased *CUP1* expression. For NMD controls, all northerns were probed with *CYH2* pre-mRNA to confirm the NMD phenotype of the yeast strains. *CYH2* pre-mRNA is a known NMD target, while *CYH2* mRNA is not (He et al. 1993). We used *SCR1* RNA as a loading control to normalize RNA levels. *SCR1* is an RNA polymerase III transcript that is not sensitive to NMD. *CYH2* pre-mRNA, *CYH2* mRNA and *SCR1* RNA are not known to be responsive to environmental copper levels. All northerns were quantified using ImageQuant software. Sigmaplot, Version 13 software was used to calculate half-lives as described in Peccarelli and Kebaara (2014).

### 3′RACE

3′RACE was used to determine the length of the 3′-UTRs as described in Kebaara et al. (2012) using the 3′RACE System for Rapid Amplification of cDNA Ends kit (Thermo Fisher Scientific, Carlsbad, CA, USA). Yeast total RNA used for steady-state northern blots was used to generate cDNA using SuperScript™II RT (Thermo Fisher Scientific, Carlsbad, CA, USA). Subsequently, the cDNA was used as the template for all primary PCR reactions. Primary PCR reactions used the Abridged Universal Amplification Primer (AUAP) from the 3′RACE kit in combination with gene-specific primers. The primary PCR product served as a template for the nested PCR reactions. All nested PCR reactions utilized gene specific primers. PCR products for both primary and nested reactions were run on 1.5% agarose gels.

### DNA methods

To create a fusion construct for *COX23* mRNA, the long 3′-UTR from *COX23* was amplified by PCR. Subsequently, the 5′-UTR and ORF of a second gene, *CYC1*, was amplified by PCR. *CYC1* mRNA is not an NMD target. Third, ligation-mediated PCR fused the two PCR fragments. To generate *CYC1COX23* 3′-UTR, the *CYC1* 5′-UTR and ORF were fused to 350 nt from the *COX23* 3′-UTR. The fusion construct was then inserted into the TOPO-TA cloning vector according to manufacturer’s instructions and sent for sequencing to confirm that the correct fusion construct was created. Next, *CYC1COX23* 3′-UTR was digested with *Bam*HI and *Sac*I before ligation into pRS425 digested with the same enzymes.

## Results

### *COX17* and *COX19* mRNAs are direct NMD targets in rich media, while *COX23* mRNA is an indirect target

**Fig. 1:**
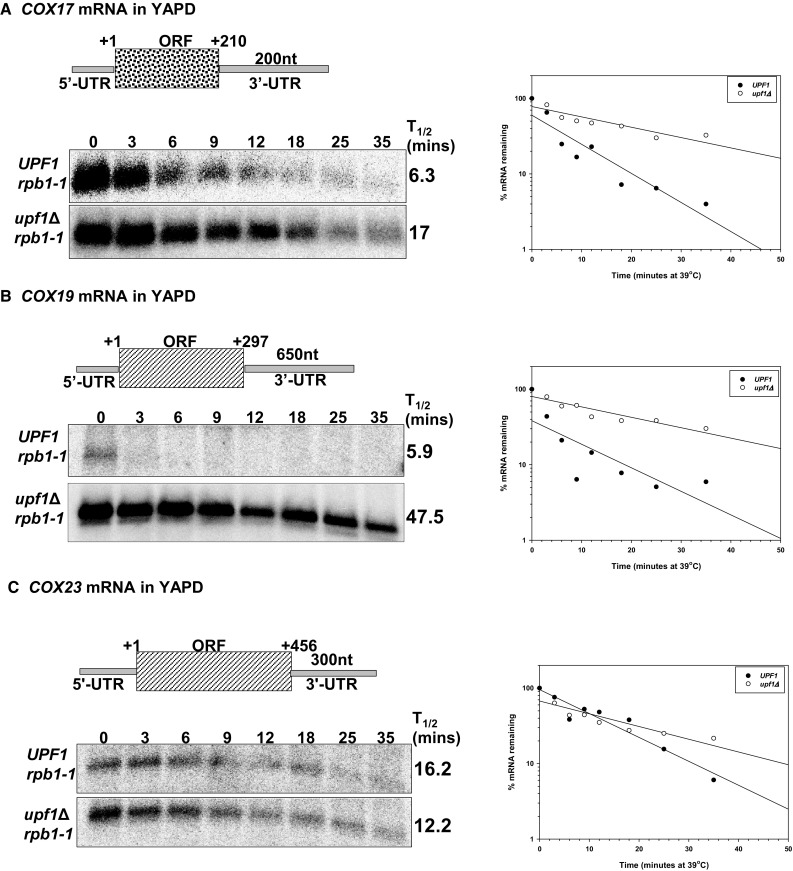
*COX17* and *COX19* mRNAs are direct NMD targets in rich media while *COX23* mRNA is an indirect target. Schematic representations of *COX17, COX19* and *COX23* mRNAs (**a**–**c**, respectively). Representative half-life northern blots were measured with total RNA extracted from wild-type strain AAY334 (*UPF1 rpb1-1*; Kebaara et al. 2003) and NMD mutant strain AAY335 (*upf1Δ rpb1-1*; Kebaara et al. 2003). Yeast cells were harvested over a 35-min period at different time points indicated above the northern blots. The northern blots were probed with radiolabeled DNA from the *COX23, COX19* and *COX17* ORFs, respectively. For controls, the membranes were probed with *CYH2* and *SCR1* DNA. The half-lives were determined using SigmaPlot graphs and are shown to the right of each northern blot. Typical SigmaPlot graphs showing the decay rate of *COX17* (**a**), *COX19* (**b**) and *COX23* (**c**) mRNAs in *UPF1* and *upf1Δ* yeast strains are shown to the right of the half-life northern blots. The half-life measurements are an average of at least three independent experiments and are calculated based on the time required for half of the original RNA to degrade

Because the proteins encoded by *COX17, COX19* and *COX23* mRNAs are homologues and function in mitochondrial copper utilization, we examined whether they are regulated by NMD similarly. In agreement with our previous study (Peccarelli et al. 2016), we found that in rich media the major *COX19* mRNA isoform is directly regulated by NMD, while in the same conditions *COX23* is indirectly regulated by the pathway (Fig. 1b, c; Table 2). Measurement of *COX17* mRNA half-life in wild-type and NMD mutant strains showed that the *COX17* mRNA is a direct NMD target in rich media (Fig. 1a). In these conditions, the half-life of *COX17* mRNA in the wild-type strain (*UPF1*) was 6.3 min relative to 17.0 min in the NMD mutant strain.

**Table 2:** *COX17, COX19* and *COX23* mRNA half-lives were measured in isogenic wild-type (*UPF1 rpb1-1*) and NMD mutants (*upf1Δ rpb1-1*)

Growth media	Yeast strain	*COX17* mRNA	*COX19* mRNA	*COX23* mRNA
YAPD	*UPF1*	6.3 ± 1.5	5.94 ± 1.44^a^	16.19 ± 2.43^a^
YAPD	*upf1∆*	17 ± 4	47.48 ± 22.51^a^	12.20 ± 2.05^a^
Low Cu	*UPF1*	6.7 ± 0.6	3.7 ± 0.6	7.7 ± 4.0
Low Cu	*upf1∆*	7.5 ± 1.0	14.7 ± 4.2	7 ± 2.6
100 µM Cu	*UPF1*	12.3 ± 4.7	4.0 ± 1.0	10.7 ± 4.5
100 µM Cu	*upf1∆*	13 ± 3.0	12.3 ± 0.57	9.6 ± 4.7

All yeast strains used were grown under the conditions indicated in the table

*ND* not determined

^a^Half-lives were reported previously (Peccarelli et al. 2016)

Since Cox17p, Cox19p and Cox23p function in CcO metallation and are regulated differentially by NMD in rich media, we examined the extent to which environmental conditions affect NMD-mediated regulation of these mRNAs. To test this, we determined the steady-state and half-lives of the three mRNAs under copper deplete and excess copper conditions.

### Differential regulation of *COX17, COX19* and *COX23* mRNAs by NMD under low copper conditions

**Fig. 2:**
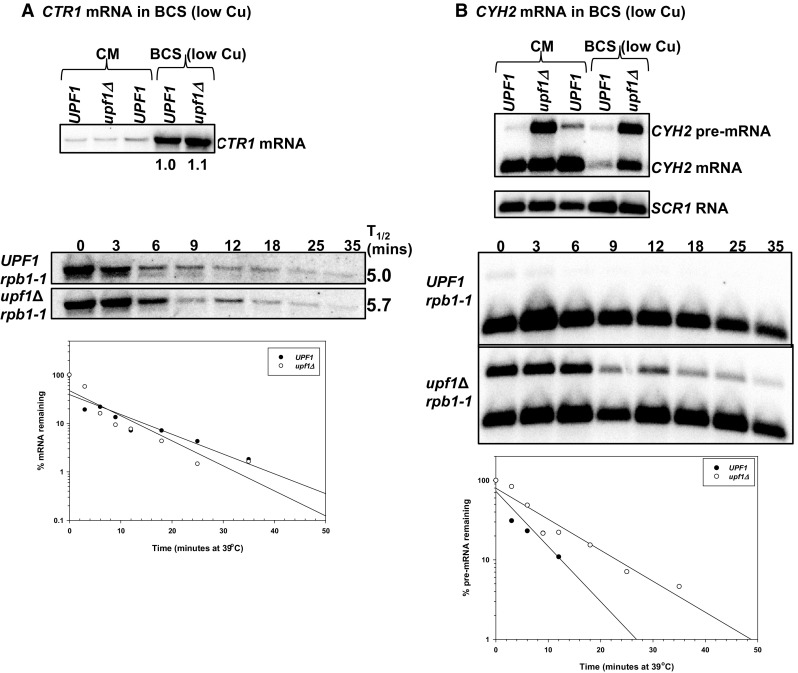
The NMD pathway is functional under low copper conditions and the *CTR1* mRNA is not regulated by NMD under these conditions. Representative steady-state mRNA accumulation levels were measured with RNA extracted from isogenic wild-type strain W303 (*UPF1*) and NMD mutant AAY320 (*upf1Δ*) yeast strains grown in complete minimal media containing bathocuproinedisulfonic acid (BCS). Half-life northern blots with total RNA extracted from wild-type strain AAY334 (*UPF1 rpb1-1*; Kebaara et al. 2003) and NMD mutant strain AAY335 (*upf1Δ rpb1-1*; Kebaara et al. 2003). Yeast cells were harvested over a 35-min time period at different time points indicated above the northern blots. The northern blots were sequentially probed with radiolabeled DNA from the *CTR1* (**a**), *CYH2* and *SCR1* (**b**). *CTR1* encodes a high-affinity copper transporter of the plasma membrane. Reduced copper levels result in increased *CTR1* mRNA expression. Typical SigmaPlot graphs showing the decay rate of *CTR1* (**a**) and *CYH2* pre-mRNA in *UPF1* and *upf1Δ* yeast strains are shown below the half-life northern blots. The half-life measurements are an average of at least three independent experiments

To investigate the effect of environmental copper levels on regulation of *COX17, COX19* and *COX23* mRNAs by NMD, we measured the steady-state and half-lives of the mRNAs in wild-type and NMD mutant yeast strains grown under low copper conditions [media containing bathocuproinedisulfonic acid (BCS)]. To confirm that low copper conditions were achieved the northern blots were first probed with *CTR1. CTR1* encodes a high-affinity copper transporter of the plasma membrane. Reduced copper levels result in increased *CTR1* expression. Under low copper conditions, *CTR1* mRNA levels were elevated in both wild-type and NMD mutants (Fig. 2a). Additionally, under these conditions the *CTR1* mRNA is not an NMD target. The half-life of *CTR1* mRNA in the wild-type strain was 5.0 min relative to 5.7 min in the NMD mutant strain (Fig. 2a, bottom panel). Furthermore, under these conditions NMD is functional as shown by the steady-state accumulation and stabilization of the *CYH2* pre-mRNA in the NMD mutants (Fig. 2b).

**Fig. 3:**
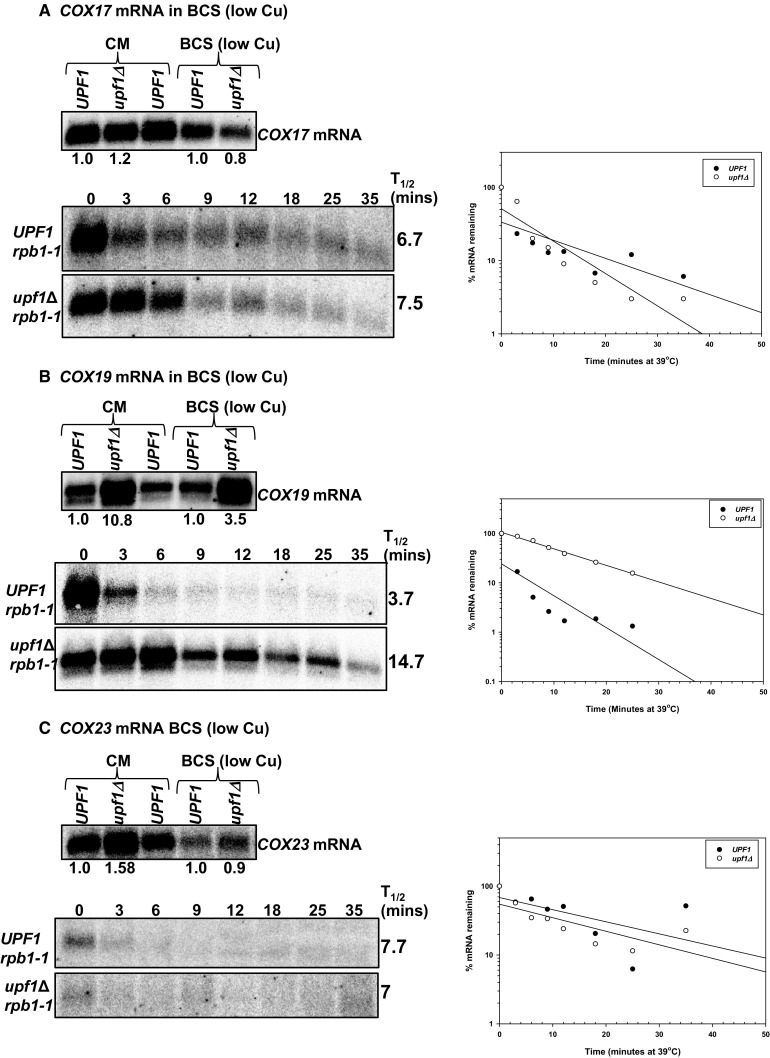
*COX23* mRNA is immune to NMD under low copper conditions; *COX17* mRNA is indirectly regulated by the pathway, while *COX19* mRNA is a direct NMD target under this conditions. Representative steady-state mRNA accumulation levels (**a**–**c**, top panels) were measured as described in Fig. 2. Half-life northern blots (**a**–**c**, bottom panels) with total RNA extracted from wild-type strain AAY334 (*UPF1 rpb1-1*; Kebaara et al. 2003) and NMD mutant strain AAY335 (*upf1Δ rpb1-1*; Kebaara et al. 2003) were measured as described in Fig. 2. The northern blots were probed with radiolabeled DNA from the *COX23, COX19*, and *COX17*, respectively. The half-lives were determined using SigmaPlot and are shown to the right of each northern blot. The half-life measurements are an average of at least three independent experiments

Under low copper conditions, one *COX17* mRNA isoform was detected on northern blots and the mRNA was not regulated by NMD. *COX17* mRNA did not accumulate to higher levels in NMD mutants and was not stabilized in cells with a non-functional NMD pathway (Fig. 3a; Table 2). In these conditions, the half-life of *COX17* mRNA in the wild-type strain (*UPF1*) was 6.7 min relative to 7.5 min in the NMD mutant (Fig. 3a; Table 2). In the wild-type strain, the half-life was similar to the observed half-life in rich media. While in the NMD mutant, the *COX17* mRNA half-life was 2.5-fold longer in rich media compared with low copper conditions (Figs. 1a, 3a).

Conversely, *COX19* mRNA accumulated to higher levels in NMD mutants and was stabilized in yeast cells with a non-functional NMD pathway (Fig. 3b; Table 2). *COX19* mRNA accumulated 3.5 (± 0.8)-fold higher in NMD mutants relative to the wild-type strain. Under these conditions, the major *COX19* mRNA isoform is degraded faster in wild-type cells relative to the NMD mutant (Fig. 3b; Table 2). The half-lives were similar in wild-type strains grown under low copper and rich media (Figs. 1b, 2b). On the other hand, *COX19* mRNA half-life is 3.2-fold longer in NMD mutants grown in rich media relative to low copper conditions (Figs. 1b, 3b).

*COX23* mRNA did not accumulate to higher levels in NMD mutants and was not stabilized in yeast cells with a non-functional NMD pathway (Fig. 2c; Table 2). *COX23* mRNA accumulated 0.9 (± 0.4)-fold higher in NMD mutants relative to wild-type cells. Under these conditions, *COX23* mRNA is degraded at comparable rates in wild-type cells relative to NMD mutants (Fig. 3c; Table 2). The half-life of *COX23* mRNA in the wild-type strain (*UPF1*) was 7.7 min relative to 7 min in the NMD mutant strain. The half-life of *COX23* mRNA was shorter in both wild-type and NMD mutant strains grown under low copper conditions relative to yeast strains grown in rich media (Figs. 1c, 3c).

Overall, under low copper conditions *COX19* mRNA accumulate to higher levels in the NMD mutants relative to the wild-type strains, while *COX17* and *COX23* mRNAs do not accumulate to higher levels (Fig. 3a–c). Interestingly, under these conditions, *COX19* mRNA is the only NMD target, while *COX23* and *COX17* mRNAs escape degradation by the pathway (Fig. 3a–c, right panels; Table 2). This differs from what we observed with rich media where *COX19* and *COX17* mRNAs are direct NMD targets.

### Under media supplemented with 100 µM copper *COX17* mRNA is immune to NMD-mediated degradation, *COX23* mRNA is indirectly regulated, while *COX19* mRNA is directly regulated by the pathway

**Fig. 4:**
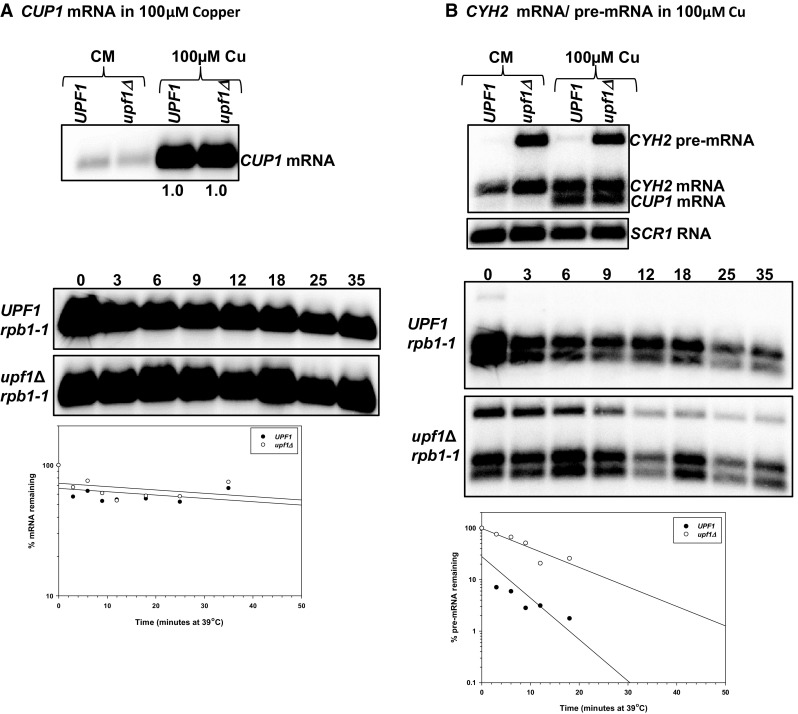
The NMD pathway is functional under 100 µM copper and the *CUP1* mRNA is not an NMD target under this conditions. Steady-state mRNA accumulation levels were measured with RNA extracted from isogenic wild-type strain W303 (*UPF1*) and NMD mutant AAY320 (*upf1Δ*) yeast strains grown in media containing 100 µM copper (top panels). Half-life northern blots with total RNA extracted from wild-type strain AAY334 (*UPF1 rpb1-1*; Kebaara et al. 2003) and NMD mutant strain AAY335 (*upf1Δ rpb1-1*; Kebaara et al. 2003) grown in media containing 100 µM copper (bottom panels). Yeast cells were harvested as described in Fig. 2 and probed with radiolabeled DNA from the *CUP1* (**a**), *CYH2* and *SCR1* (**b**). *CUP1* encodes a metallothionein that binds copper. The bottom band on the *CYH2* mRNA/pre-mRNA steady-state and half-live northern is the *CUP1* mRNA, which is highly overexpressed under high copper conditions. The *CUP1* gene is induced by the Ace1 transcription factor when yeast cells are exposed to elevated copper levels. Increases in copper levels result in increased *CUP1* expression. Half-lives were determined using SigmaPlot, by measuring the time it takes for half of the original mRNA levels to degrade. The half-life graphs are shown below each half-life northern blot. The half-life measurements are an average of at least three independent experiments

To further investigate the extent to which regulation of *COX17, COX19* and *COX23* mRNAs by NMD is dependent on environmental copper levels, we examined the regulation of the mRNAs in wild-type and NMD mutant yeast strains grown in excess copper (media containing 100 µM copper). The elevated copper conditions were verified by probing the northern blots with *CUP1* mRNA. *CUP1* encodes a metallothionein that binds copper. The *CUP1* gene is induced by the Ace1 transcription factor when yeast cells are exposed to elevated copper levels. Increases in copper levels result in increased *CUP1* expression. *CUP1* mRNA accumulated 4.25 (± 0.5)-fold higher in wild-type yeast strains grown under high copper conditions relative to yeast strains grown in regular copper levels. Additionally, *CUP1* mRNA accumulated 14.33 (± 1.4)-fold higher in NMD mutant strains grown under elevated copper levels (Fig. 4a, left panel). Comparing *CUP1* mRNA levels in wild-type and NMD mutants shows that the mRNA does not accumulate in the NMD mutant under normal or elevated copper conditions (Fig. 4a, left panel). Thus, *CUP1* mRNA is not an NMD target in rich media or under elevated copper conditions. *CUP1* mRNA half-life could not be determined within the time points utilized in these experiments, the mRNA did not decay within the 35-min time period in either wild-type or NMD mutant yeast strains. It appears that, under elevated copper levels *CUP1* mRNA is very stable and not regulated by the NMD pathway (Fig. 4a, left panel).

Furthermore, the NMD pathway is functional under high copper conditions as validated by *CYH2* pre-mRNA and mRNA steady-state accumulation and half-lives in excess copper conditions (Fig. 4b). We tested the NMD status of yeast cells grown under elevated copper levels because excess copper can induce stress (like hypoxia and ER stress). Stress has been shown to inhibit NMD.

**Fig. 5:**
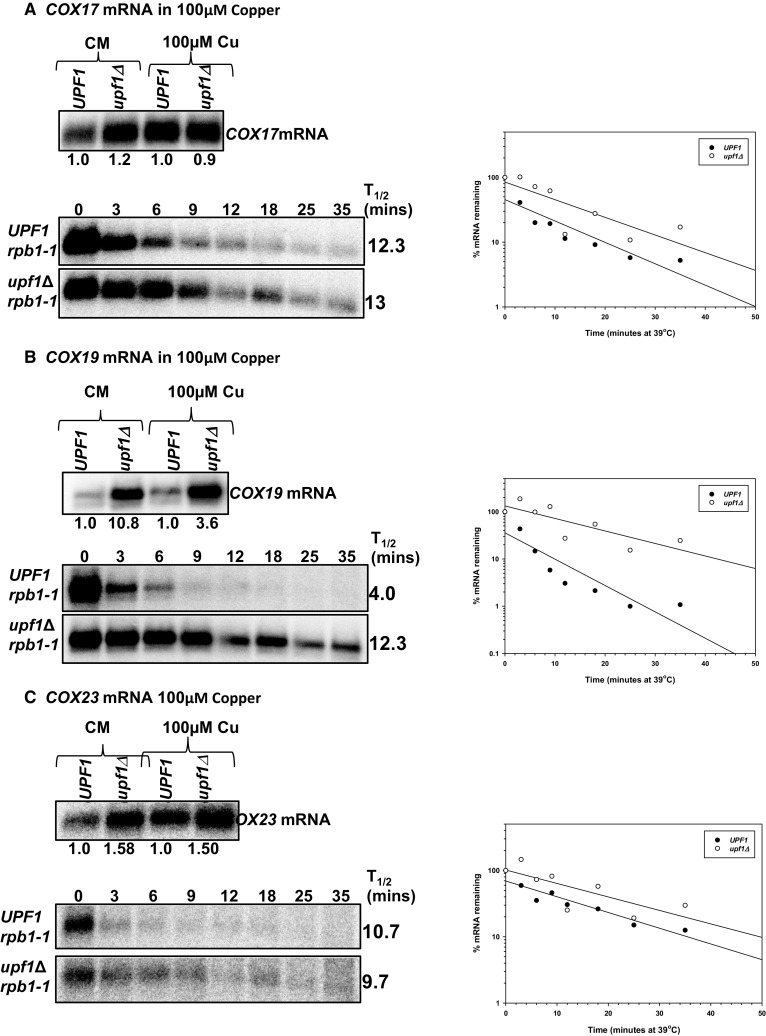
*COX19* mRNA is a direct NMD target under high copper conditions, while *COX23* mRNA is an indirect target and *COX17* mRNA is immune to degradation by the pathway. Representative steady-state mRNA accumulation levels (**a**–**c**, top panels) were measured with RNA as described in Fig. 4. Half-life northern blots (**a**–**c**, bottom panels) with total RNA extracted from wild-type strain AAY334 (*UPF1 rpb1-1*; Kebaara et al. 2003) and NMD mutant strain AAY335 (*upf1Δ rpb1-1*; Kebaara et al. 2003). Yeast cells were harvested as described in Fig. 4 and probed with radiolabeled DNA from the *COX23, COX19*, and *COX17* ORFs, respectively. Half-lives were determined using SigmaPlot, by measuring the time it takes for half of the original mRNA levels to degrade. The half-life graphs are shown to the right of each half-life northern blot. The half-life measurements are an average of at least three independent experiments

Under elevated copper levels, one *COX17* mRNA isoform was detected and the mRNA was not regulated by NMD. *COX17* mRNA did not accumulate to higher levels in NMD mutants and was not stabilized in yeast cells with a non-functional NMD pathway (Fig. 5a; Table 2). The half-life of *COX17* mRNA in the wild-type strain was 12.3 min relative to 13 min in the NMD mutant strain. In wild-type strains, *COX17* mRNA half-life was longer under elevated copper conditions relative to both low copper conditions and rich media. In NMD mutants, *COX17* mRNA half-life was comparable in rich media and elevated copper levels but faster under low copper conditions (Fig. 5a; Table 2). These *COX17* mRNA half-life observations are similar to low copper but unlike what we observed in rich media (Figs. 1a, 3a).

In addition, one major *COX19* mRNA isoform was detected in wild-type and NMD mutant strains grown in media supplemented with 100 µM copper. The major *COX19* mRNA isoform accumulated to higher levels in NMD mutants. Steady-state accumulation levels of *COX19* mRNA were threefold higher in wild-type and NMD mutant strains grown in complete minimal media relative to complete minimal media supplemented with 100 µM copper. Under these conditions, *COX19* mRNA was degraded faster in wild-type cells relative to NMD mutants. The half-life of *COX19* mRNA in the wild-type strain was 4.0 min relative to 12.3 min in the NMD mutant strain. This was comparable to what we observed with rich media and under low copper (Fig. 5b; Table 2).

Further, one *COX23* mRNA isoform was detected in wild-type and NMD mutant strains grown on media containing 100 µM copper. Steady-state accumulation levels of *COX23* mRNA were comparable in wild-type (*UPF1*) and NMD mutant (*upf1Δ*) strains grown in complete minimal or complete minimal supplemented with 100 µM copper. Furthermore, under these conditions, the *COX23* mRNA was an indirect NMD target (Fig. 5c; Table 2). The half-life of *COX23* mRNA in the wild-type strain was 10.7 min relative to 9.7 min in the NMD mutant strain. These observations are comparable to rich media where we identified *COX23* as an indirect NMD target (Table 2). The indirect regulation of *COX23* mRNA by NMD under several environmental copper levels promoted us to examine the functionality of the NMD targeting feature within *COX23* mRNA.

### The *COX23* mRNA 3′-UTR is sufficient to target an NMD-insensitive transcript for NMD-mediated degradation

**Fig. 6:**
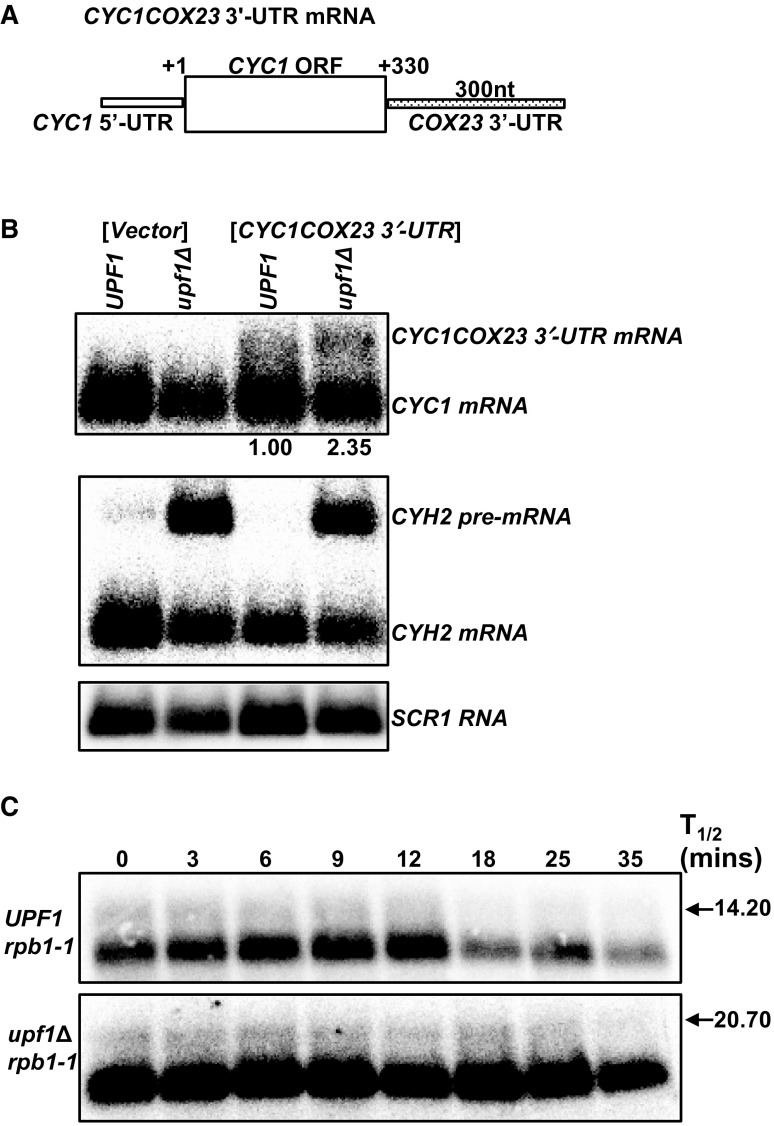
The *COX23* 3′-UTR is sufficient to target *CYC1* mRNA to NMD. Schematic representation of *CYC1COX23* 3′-UTR mRNA (**a**) and representative northern blots of the *CYC1COX23* 3′-UTR steady-state mRNA accumulation levels (**b**) and half-lives (**c**). The northern blots were probed with DNA specific to the 5′-UTR and ORF of *CYC1*. All yeast cells for **b** and **c** were grown in synthetic complete media lacking leucine and were harvested as described in Fig. 1. Steady-state and half-life mRNA measurements are an average of at least three independent experiments. The half-lives were determined using SigmaPlot. *CYH2* and *SCR1* are controls

Of the three mRNAs encoding proteins involved in metallation of CcO and regulated in an NMD-dependent manner, *COX23* mRNA has an identifiable NMD targeting feature and is immune to degradation or indirectly regulated by the pathway in the conditions tested here. *COX23* mRNA is an indirect NMD target in rich media and under elevated copper conditions (Figs. 1c, 5c) (Peccarelli et al. 2016). *COX23* mRNA has an atypically long 3′-UTR of 300 nt that does not appear to target the mRNA to NMD-mediated degradation. These observations are distinct from the other two mRNAs encoding functionally homologous proteins including *COX19. COX19* mRNA has an atypically long 3′-UTR and was found to be directly regulated by NMD under all three conditions tested here and previously (Figs. 1b, 3b, 5b) (Peccarelli et al. 2016). Furthermore, the 3′-UTR of *COX19* plays a role in the regulation of this mRNA by NMD (Peccarelli et al. 2014, 2016). It is possible that the *COX23* mRNA is an indirect NMD target in select conditions because the mRNAs 3′-UTR is in an incorrect context. Alternatively, this NMD targeting feature could target the *COX23* mRNA to NMD in specific environmental conditions not tested here, as was previously observed with *MAC1* mRNA (Peccarelli et al. 2016). To investigate if the *COX23* 3′-UTR is sufficient to target an NMD-insensitive mRNA to the pathway in defined media, we generated the *CYC1COX23* 3′-UTR fusion mRNA. The fusion mRNA contains the 5′-UTR and ORF from *CYC1* fused to the *COX23* 3′-UTR (Fig. 6a). The *CYC1* mRNA, which encodes for iso-1-cytochrome *c*, was used because it has previously been utilized to study instability elements and is insensitive to the NMD pathway (Zaret and Sherman 1984; Peccarelli et al. 2014).

The *CYC1COX23* 3′-UTR mRNA was significantly less abundant than the endogenous *CYC1* mRNA and accumulated 2.35 (± 0.68)-fold higher in the NMD mutants relative to the wild-type yeast strain (Fig. 6b). This observation suggests that the *COX23* mRNA 3′-UTR contains a general mRNA destabilizing element. Additionally, the half-life of the *CYC1COX23* 3′-UTR mRNA in the wild-type strain was 14.20 ± 5.50 min relative to 20.70 ± 3.50 min in the NMD mutant strain (Fig. 6c). Although the values were somewhat close, the difference between the two half-lives is statistically significant, indicating that *CYC1COX23* 3′-UTR fusion mRNA is directly regulated by NMD. These results suggest that the *COX23* 3′-UTR is sufficient to target an NMD-insensitive transcript to the pathway and supports the conclusion that the *COX23* mRNA evades direct regulation by NMD in specific conditions.

### NMD mutants’ respiratory impairments on non-fermentable carbon sources is recovered by elevated copper levels

Previous studies have found that NMD mutants have respiratory impairments when grown on non-fermentable carbon sources. This growth defect could be due to accumulation of products interfering with respiratory function or altered expression of mRNAs involved in mitochondrial copper homeostasis. To test the extent to which NMD-mediated regulation of mRNAs involved in mitochondrial copper utilization affects this respiratory impairment, we grew wild-type and NMD mutant yeast strains on media containing lactate, a non-fermentable carbon source and supplemented the media with excess copper.

**Fig. 7:**
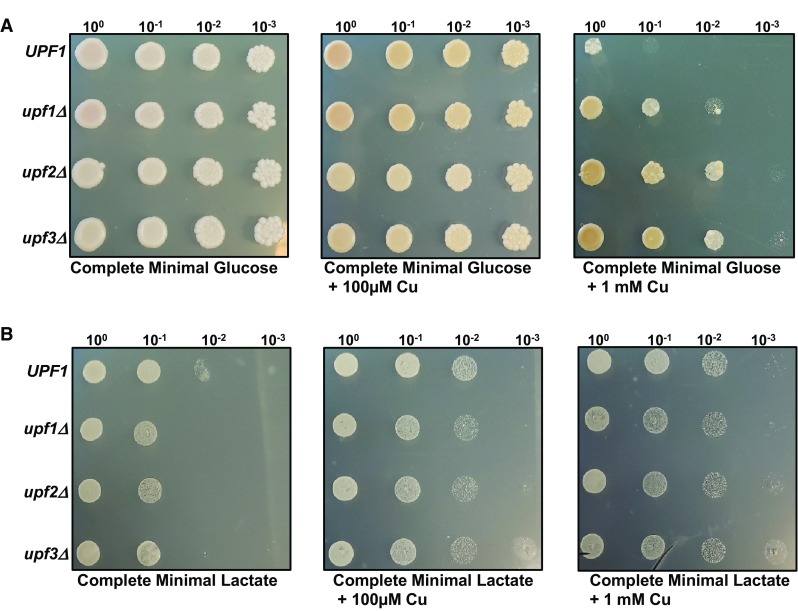
NMD mutants’ respiratory impairments on non-fermentable carbon sources is recovered by elevated copper levels. *UPF1* (W303a), *upf1Δ* (AAY320), *upf2Δ* (HFY1300) and *upf3Δ* (HFY861) yeast cells were grown to mid-log phase on complete minimal media. Tenfold serial dilutions of the cells were spotted onto complete minimal medium with either glucose (**a**) or lactate (**b**) as the carbon source. Additionally, the four yeast strains were spotted on media containing either 100 µM copper (middle panels) or 1 mM copper (right panels) and incubated 30 °C for 3 days

**Fig. 8:**
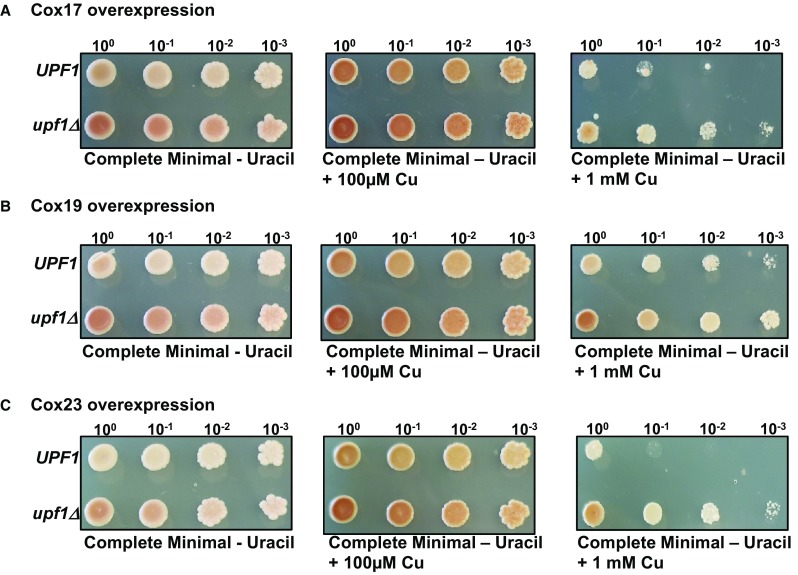
Overexpression of *COX19* enhances growth of wild-type yeast strains on media containing elevated copper levels. *UPF1* (W303a) and *upf1Δ* (AAY327) yeast strain were transformed with pGT74/T3 (**a**), pG188/T1 (**b**) and pG84/T1 (**c**) plasmids and grown to mid-log phase on complete minimal media lacking uracil. Tenfold serial dilutions of the cells were spotted onto complete minimal medium lacking uracil (left panels), containing either 100 µM copper (middle panels) or 1 mM copper (right panels) and incubated 30 °C for 4 days

As we previously reported, NMD mutants are more tolerant of toxic copper levels when grown on glucose as a carbon source. This copper tolerance phenotype is clearly observed when wild-type and NMD mutants are grown on media containing 1 mM copper. When wild-type, and NMD mutants were grown on media containing lactate as the carbon source, the NMD mutants had impaired growth (Fig. 7b, left panel). *upf1Δ, upf2Δ*, and *upf3Δ* mutants all had equally reduced growth. Addition of excess copper to media containing lactate resulted in enhanced growth of all the strains predominantly the NMD mutants. Interestingly, *upf2Δ* and *upf3Δ* NMD mutant strains showed noticeably enhanced growth compared to the wild-type strain on media containing lactate with elevated copper (Fig. 7b, right panel). We also examined the effect overexpressing *COX17, COX19* and *COX23* has on wild-type and NMD mutants’ strains grown on media containing elevated copper levels. Overexpression of *COX17* and *COX23* resulted in a phenotype similar to wild-type and NMD mutant yeast strains. The NMD mutant strain was more tolerant of elevated copper levels (Fig. 8a, c). Interestingly, overexpression of *COX19* results in increased tolerance of the wild-type strain to elevated copper levels (Fig. 8b). Notably, *COX19* mRNA is the only mRNA that was regulated by NMD in all the conditions tested here including elevated copper conditions. Thus, it appears that regulation of mRNAs involved in mitochondrial copper utilization by NMD affects growth of wild-type and NMD mutants on a non-fermentable carbon sources.

## Discussion

The observation that in some conditions the NMD pathway differentially regulates mRNAs that encode structurally and functionally related proteins suggests that the regulation of these mRNAs maybe condition specific. This form of regulation would allow yeast cells to control the expression of specific mRNAs in response to environmental changes. Since these mRNAs encode proteins involved in mitochondrial copper utilization, copper levels in the environment could control the regulation of the mRNAs by NMD.

*COX17* mRNA has been studied extensively and shown to be regulated by puf3 protein depending on carbon source (Miller et al. 2014). *COX23* and *COX19* mRNAs are less well understood. Cox17p, Cox19p and Cox23p are essential for CcO assembly because they are required for mitochondrial copper utilization. Copper is an essential cofactor in the mitochondrial electron transport chain. We report here that the three mRNAs are differentially regulated by NMD depending on environmental copper levels. The differential regulation of the mRNAs by the pathway may have functional significance to yeast cells.

For example, *COX17* mRNA is directly regulated by NMD only in rich media but not under low or high copper conditions. It could be that under adequate copper conditions, NMD maintains *COX17* mRNA at the appropriate levels for mitochondrial copper utilization. Under low or high copper conditions, *COX17* mRNA levels could be adjusted. Additionally, *COX17* mRNA has a short ORF of 210 nucleotides. This may well activate NMD-mediated degradation of the mRNA in rich media because the only other recognizable NMD targeting feature is possible leaky scanning that could promote frameshifting and NMD (Celik et al. 2017). As mentioned above, *COX17* mRNA decay is regulated by puf3 protein (Olivas and Parker 2000). puf3 protein binds primarily to nuclear encoded mRNAs that encode proteins with mitochondrial function. Thus, in rich media *COX17* mRNA is regulated by puf3 protein and the NMD pathway. Interestingly of the mRNAs investigated here, *COX17* and *COX23* mRNAs were found to associate with puf3 protein and *COX19* mRNA has not been reported to be regulated by puf3 (Gerber et al. 2004; Foat et al. 2005).

On the other hand, the main *COX19* mRNA isoform was found to be a direct NMD target under all of conditions tested. The half-life of *COX19* mRNA was almost twice as long in wild-type yeast strains grown in rich media relative to low copper or excess copper conditions. This observation suggests that additional *COX19* mRNA might be required to translate more Cox19p to support the metallation of CcO in rich media. Interestingly, overexpression of *COX19* resulted in wild-type yeast cells that are more tolerant of toxic copper levels. This observation suggests that precise regulation of *COX19* mRNA by NMD under diverse conditions is required to maintain precise mitochondrial copper homeostatic mechanisms. In addition, *COX19* mRNA has a long 3′-UTR that contributes to the degradation of the mRNA by NMD (Peccarelli et al. 2016).

Interestingly both *COX19* and *COX23* mRNA have identical NMD targeting features but are differentially regulated by NMD. *COX23* mRNA is immune to NMD under low copper conditions and is an indirect NMD target in rich media and high copper. However, *COX23* mRNA accumulates to higher levels in these conditions; this indicates that there are other factors upstream of *COX23* mRNA that regulate the mRNA levels in response to NMD. This suggests that the NMD pathway, based on environmental conditions, may differentially regulate homologous mRNAs with identical NMD targeting features. Furthermore, because the *COX19* mRNA 3′-UTR is twice as long as the *COX23* mRNA3′-UTR, it could be that the *COX19* 3′-UTR is a more efficient NMD targeting feature compared to the *COX23* 3′-UTR.

However, we found that the *COX23* 3′-UTR is sufficient to target an NMD-insensitive mRNA to the pathway. This observation shows that the *COX23* mRNAs 3′-UTR is not too short to directly target the mRNA to the NMD pathway. It is possible that the endogenous *COX23* mRNA’s 3′-UTR is in the incorrect context or that the mRNA is directly regulated by NMD in specific environmental conditions. As mentioned above, we previously found that an additional mRNA belonging to this general functional group, *MAC1* mRNA, was directly regulated by NMD in rich media but not under low copper conditions (Peccarelli et al. 2016). We hypothesize that a feature within the 5′-UTR or ORF of *COX23* can stabilize the transcript. Alternatively, factors present in specific conditions can bind to the *COX23* mRNA and stabilize it. Further investigation into how *COX23* mRNA evades direct regulation by NMD in rich media, and high copper and the extent to which additional environmental conditions lead to direct NMD targeting of *COX23* mRNA will provide insights into the targeting of functional groups of mRNAs.

Regulation of mRNAs involved in mitochondrial copper utilization by NMD has physiological consequences to yeast cells. Expression of Cox17, Cox19 and Cox23 proteins are required for respiratory capability (Longen et al. 2009). Furthermore, NMD mutants have impaired growth on non-fermentable carbon sources. Additional copper in the growth media can enhance the impaired growth defect of the NMD mutants. This suggests that imbalance in mitochondrial copper homeostasis is partly responsible for the NMD mutants’ respiratory defect. Interestingly, additional copper can recover impaired growth of a *COX17* mutant but not a *COX19* mutant. Additionally, extra copper can also enhance the growth of NMD mutants grown on non-fermentable carbon sources. This observation suggests that misregulation of mitochondrial copper homeostatic mRNAs is partly responsible for the growth defect. This study adds to reports demonstrating that mitochondrial gene expression is regulated at the RNA level. Specifically long noncoding RNAs have been reported to regulate mitochondrial gene expression (De Paepe et al. 2018). In summary, the studies reported here show mitochondrial copper homeostatic mechanisms occur at the mRNA level via the NMD pathway.

## Conclusions

In this study, we showed that: (1) *COX17* mRNA is a direct NMD target under specific growth conditions; (2) *COX17, COX19* and *COX23* mRNAs are differentially regulated by NMD based on environmental copper levels; (3) we determined that the *COX23* 3′-UTR is sufficient to trigger NMD; (4) growth impairment of wild-type and NMD mutants on non-fermentable carbon source is enhanced by excess copper; (5) overexpression of *COX19* enhances growth of wild-type yeast strains on toxic amounts of copper. This study as well as our previous studies suggests that regulation of functionally related mRNAs by NMD may be dependent on environmental conditions and can be differential. Our study also provides insight into the mechanism NMD plays in regulating natural mRNAs.
